# HIV specific CD8^+^ T_RM_-like cells in tonsils express exhaustive signatures in the absence of natural HIV control

**DOI:** 10.3389/fimmu.2022.912038

**Published:** 2022-10-18

**Authors:** Rabiah Fardoos, Sarah K. Nyquist, Osaretin E. Asowata, Samuel W. Kazer, Alveera Singh, Abigail Ngoepe, Jennifer Giandhari, Ntombifuthi Mthabela, Dirhona Ramjit, Samita Singh, Farina Karim, Søren Buus, Frank Anderson, J. Zachary Porterfield, Andile L. Sibiya, Rishan Bipath, Kumeshan Moodley, Warren Kuhn, Bonnie Berger, Son Nguyen, Tulio de Oliveira, Thumbi Ndung’u, Philip Goulder, Alex K. Shalek, Alasdair Leslie, Henrik N. Kløverpris

**Affiliations:** ^1^ Africa Health Research Institute (AHRI), Durban, South Africa; ^2^ Department of Immunology and Microbiology, University of Copenhagen, Copenhagen, Denmark; ^3^ Institute for Medical Engineering & Science, Department of Chemistry, and Koch Institute for Integrative Cancer Research, Massachusetts Institute of Technology, Cambridge, MA, United States; ^4^ Program in Computational and Systems Biology, Massachusetts Institute of Technology, Cambridge, MA, United States; ^5^ KwaZulu-Natal Research Innovation and Sequencing Platform (KRISP), Nelson R Mandela School of Medicine, University of KwaZulu-Natal, Durban, South Africa; ^6^ Discipline of General Surgery, Inkosi Albert Luthuli Central Hospital, University of KwaZulu-Natal, Durban, South Africa; ^7^ Department of Otolaryngology-Head & Neck Surgery, Division of Infectious Diseases, University of Kentucky, Lexington, KY, United States; ^8^ Department of Microbiology, Immunology and Molecular Genetics, - Division of Infectious Diseases, University of Kentucky, Lexington, KY, United States; ^9^ Department of Internal Medicine - Division of Infectious Diseases, University of Kentucky, Lexington, KY, United States; ^10^ Department of Otorhinolaryngology & Head & Neck Surgery, Inkosi Albert Luthuli Central Hospital, University of KwaZulu-Natal, Durban, South Africa; ^11^ Department of Otorhinolaryngology, King Edward VIII hospital, University of KwaZulu-Natal, Durban, South Africa; ^12^ Department of Ear Nose and Throat, General Justice Gizenga Mpanza Regional Hospital (Stanger Hospital), University of KwaZulu-Natal, Durban, South Africa; ^13^ Computer Science & Artificial Intelligence Lab and Department of Mathematics, Massachusetts Institute of Technology, Cambridge, MA, United States; ^14^ HIV Pathogenesis Programme, The Doris Duke Medical Research Institute, University of KwaZulu Natal, Durban, South Africa; ^15^ University College London, Division of Infection and Immunity, London, United Kingdom; ^16^ Department of Paediatrics, University of Oxford, Oxford, United Kingdom; ^17^ Broad Institute of Massachusetts Institute of Technology and Harvard, Cambridge, MA, United States; ^18^ Ragon Institute of MGH, Harvard, Cambridge, MA, United States

**Keywords:** CD8^+^ T_RM_ cells, HIV, PD-1, tonsils, exhaustion, natural HIV control

## Abstract

Lymphoid tissues are an important HIV reservoir site that persists in the face of antiretroviral therapy and natural immunity. Targeting these reservoirs by harnessing the antiviral activity of local tissue-resident memory (T_RM_) CD8^+^ T-cells is of great interest, but limited data exist on T_RM_-like cells within lymph nodes of people living with HIV (PLWH). Here, we studied tonsil CD8^+^ T-cells obtained from PLWH and uninfected controls from South Africa. We show that these cells are preferentially located outside the germinal centers (GCs), the main reservoir site for HIV, and display a low cytolytic and a transcriptionally T_RM_-like profile distinct from blood CD8^+^ T-cells. In PLWH, CD8^+^ T_RM_-like cells are expanded and adopt a more cytolytic, activated, and exhausted phenotype not reversed by antiretroviral therapy (ART). This phenotype was enhanced in HIV-specific CD8^+^ T-cells from tonsils compared to matched blood suggesting a higher antigen burden in tonsils. Single-cell transcriptional and clonotype resolution showed that these HIV-specific CD8^+^ T-cells in the tonsils express heterogeneous signatures of T-cell activation, clonal expansion, and exhaustion ex-vivo. Interestingly, this signature was absent in a natural HIV controller, who expressed lower PD-1 and CXCR5 levels and reduced transcriptional evidence of T-cell activation, exhaustion, and cytolytic activity. These data provide important insights into lymphoid tissue-derived HIV-specific CD8^+^ T_RM_-like phenotypes in settings of HIV remission and highlight their potential for immunotherapy and targeting of the HIV reservoirs.

## Introduction

No treatment for HIV infection is currently available to eradicate viral reservoirs, except anecdotal reports involving immune ablation followed by adoptive transfer of non-susceptible target cells (1, 2). A key challenge is the presence of these viral reservoirs within human tissues, primarily within the germinal center (GC) of lymphoid tissue structures (3, 4), which persist despite highly effective antiretroviral treatment (ART) (5–9). Even when ART is initiated during the acute phase of infection (10, 11), these reservoirs can still fuel rapid rebound of plasma viremia during treatment interruption (12). Therefore, HIV treatment will continue to require life-long ART, unless therapeutics can be developed to purge virus from these sanctuary sites. A potential to achieve this goal is to enhance pre-existing HIV-specific CD8^+^ T-cell responses within these sites and thereby facilitating the eradication of the viral reservoir. However, this requires a better understanding of the CD8^+^ T-cells present in HIV infected lymphoid tissues.

Tissue resident memory (T_RM_) CD8^+^ T-cells (13) are retained within tissues, including lymphoid tissues, and characterised by the expression of CD69, αE integrin (CD103) and absence of sphingosine-1-phosphate receptor (S1PR1) (14–17). CD8^+^ T_RM_ cells are involved in the early response to pathogens (18, 19) and initiate rapid defence upon reinfection (20). CD8^+^ T-cells mediate viral control and protect from disease progression in HIV and simian immunodeficiency virus (SIV)-infected rhesus macaque and play a significant role in restricting viral reservoirs within lymphoid tissue (21–23) also during ART (4, 24). However, in the vast majority of cases, viral control eventually fails in the absence of ART, and CD8^+^ T-cell function becomes impaired (25–27).

One obstacle for optimal CD8^+^ T-cell function and their ability to clear viral reservoirs may be related to elevated expression of programmed cell death protein 1 (PD-1) and other co-inhibitory molecules (28–30). In the blood, high levels of antigen exposure during chronic untreated HIV infection lead to increased CD8^+^ T-cell activation, terminal differentiation and ultimately dysfunction termed ‘exhaustion’ (31–36), which is directly linked to cognate epitope availability (32, 37, 38). However, PD-1 expression is elevated on T-cell subsets within tissue sites such as lymphoid organs, and may not directly relate to immune exhaustion (39). In addition, HIV-specific CD8^+^ T-cells identified in tissue typically lack the GC homing receptor CXCR5 expression (3, 40, 41), which may limit their ability to clear HIV reservoirs from the immune privileged B-cell follicles (42).

Most studies of T-cell function in people living with HIV (PLWH) are of cells derived from the blood, which do not capture the phenotype and function of CD8^+^ T_RM_ located near the viral reservoirs within lymphoid tissues (3, 4, 8). Recent studies show important differences between HIV-specific CD8^+^ T-cells located in blood and lymphoid tissues, and highlight the importance of better defining the functionality of the cells in closest proximity to viral reservoirs (39, 43–45). In particular, little is known about the expression of inhibitory receptors, such as PD-1, on HIV specific CD8^+^ T-cells and the functional significance of these markers within HIV infected tissue sites (17, 39) and is important for strategies seeking to enhance antiviral activities in HIV-infected tissue.

Here, we studied CD8^+^ T-cells in blood and palatine tonsils, a lymphoid tissue organ in the oral-pharyngeal mucosa (46), from PLWH recruited from HIV endemic areas in South Africa. We combined *in situ* phenotyping, flow cytometry and applied paired single cell RNA (scRNA) and T-cell receptor (TCR) sequencing on bulk and antigen specific CD8^+^ T-cells (HIV and CMV) to determine the impact of HIV in this compartment. We show that HIV infection increases PD-1 expression along with canonical CD69 and CD103 T_RM_ markers on tonsil CD8^+^ T-cells that is not reversed by treatment. The HIV-specific CD8^+^ T_RM_-like cells are heterogenous in transcriptional signatures of activation and exhaustion, which is linked to detectable tonsil HIV-p24 antigen and enriched in expanded HIV-specific TCR-clonotypes. Interestingly, in one natural HIV controller the HIV-specific CD8^+^ T_RM_-like cells express reduced levels of PD-1, increased follicle homing chemokine receptor CXCR5 and reduced cytolytic T-cell activity, suggesting that natural HIV controllers harbour distinct CD8^+^ T_RM_-like function within lymphoid tissue (44).

## Materials and methods

### Study participants

Human tonsils tissue samples and peripheral blood were collected from individuals being HIV^-^ (n=20), HIV^+^ chronic and naïve to ART (n=8), HIV^+^ chronic on ART (n=15). Tonsil tissue was obtained from patients undergoing routine tonsillectomy at Stanger Hospital, KwaDukuza and Addington Hospital in Durban, KwaZulu-Natal. Frozen peripheral blood samples HIV^-^ (n=10) were obtained from the Females Rising through Empowerment Support and Health (FRESH) cohort from Umlazi, Durban (Ndlovu et al., 2015). Due to the lack of obtaining matched blood from individuals undergoing routine tonsillectomy, we added more blood samples to compare with tonsil tissue, a distinct group of frozen peripheral blood samples HIV^+^ naïve to ART (n=5) and on ART (n=5) were obtained from the ‘GATEWAY’ based at the Prince Mshiyeni Memorial Hospital, Umlazi, Durban.

All participants provided informed consent. Informed consent from underaged participants was approved by a parent or guardian. The study was approved by the respective institutional review boards including the Biomedical Research Ethics Committee of the University of Kwazulu-Natal in Durban, South Africa.

### Sample processing-blood and tonsils

Blood samples were processed from fresh peripheral blood mononuclear cells (PBMCs) purified using Ficoll separation. Samples were thawed in DNase-containing (25 units ml^-1^) R10 (Sigma-Aldrich) at 37°C. Cells were rinsed at rested at 37°C for a minimum of 1 h before undergoing red-blood-cell lysis by 5-10 ml RBC lysis solution (Qiagen) for 20 min at room temperature. Cells were then stained with the appropriate antibody panel described in Flow cytometry.

Tonsil samples were processed from fresh tissue immediately after surgery. Resected tissues were washed with cold HBSS (Sigma-Aldrich) and dissected into smaller pieces. Tissues were rinsed again and resuspended in 10 ml R10, containing DNase (1 µl ml^-1^) and collagenase (4 µl ml^-1^), and disassociated in a GentleMACS dissociator (Miltenyi Biotec). Cells were rested in a shaking incubator at 37°C for 30 min and then further processed in the GentleMACS dissociator. After further resting (30 min at 37°C) and washing steps, cells were strained through a 70-µm cell strainer and washed on the final time. Cells were lysed using 5-10 ml RBC lysis buffer (Qiagen) and stained for flow cytometry analysis. In the relevant experiments, tetramer was added for 30 minutes at room temperature after resting. For identifying HIV and CMV-specific CD8^+^ T-cells.

### Flow cytometry

For FACS analysis, different antibody panels for phenotype and intracellular cytokine staining (ICS) were used. A complete list of antibodies used with identifier and source information can be found in the **Supplementary Table S14**.

Cryopreserved PBMC and TMC were thawed and rested in RPMI-1640 media (20% fetal calf serum (FCS), 1% penicillin/streptomycin, 1% L-glutamine) for 2 hours at 37°C incubator. In the relevant experiments, tetramer was added for 30 minutes at room temperature after resting for identifying HIV and CMV-specific CD8^+^ T-cells. All Samples were further surface stained including a near-infrared live/dead cell viability cell staining kit (Invitrogen) at room temperature for 20 min. Cells were then washed with PBS and acquired using the BD FACS ARIA FUSION (BD Bioscience). For experiments involving ICS, the cells were stimulated with SEB, in the presence of Golgiplug and Golgistop (BD Biosciences) for 6 hours in 37°C incubator. Cells were stained with fluorochrome-conjugated monoclonal antibodies and subsequently fixed, permeabilized and stained by BD Cytofix/cytoperm kit (BD Biosciences). Peptide-MHC tetramers were acquired from ImmunAware, Copenhagen, Denmark and stained during cell surface antibody labelling. Blocking with 20% goat serum for 20 min was done prior to intracellular antibody staining. After staining, cells were washed and fixed in 2% paraformaldehyde before acquisition on a 4 laser, 17 parameter BD FACSAria Fusion flow cytometer. Data were analyzed with FlowJo software (version 10.4.2, TreeStar).

### Fluorescent immunohistochemistry

Multiplex fluorescent immunohistochemistry experiment was performed using the Opal™ 4-Color Manual IHC kit (PerkinElmer) according to the manufacturer’s instructions. Briefly, tonsil tissue samples fixed with 4% formalin for a minimum of 48 hours were paraffin-embedded. Exactly 4μm sections were cut, deparaffinized and stained with the following unlabelled primary antibodies: CD8 (clone: C8/144B, Dako), CD4 (clone: 4B12, Dako), p24 (clone: Kal-1, Dako), CXCR5 (clone: MU5UBEE, Thermofisher Scientific), GZMB (clone: 23 H8L20, Thermofisher Scientific), CD103 (clone: NBP1-88142, Novus Biologicals) and CD69 (clone: 15B5G2, Novus Biologicals). Opal fluorophores: FITC (Opal520) was used for p24 and CD103; Texas-Red (Opal570) was used for CD8 and CD4; then Cy5 (Opal690) was used for CD8, CXCR5, Granzyme-B and CD69 signal generation in the different IHC experiments performed. DAPI was used as the nuclear counterstain. Images were acquired on a Zeiss Axio Observer Z1 inverted microscope (Olympus) and analysed with TissueFAXS imaging and TissueQuest quantitation software (TissueGnostics).

### Single-cell RNA-seq using Seq-Well S^3^

After obtaining single-cell suspension from fresh biopsies, we used the Seq-Well S^3^ platform. Full methods of implementation of this platform are described (47, 48). Briefly, 15,000 cells in 200 mL RPMI + 10% FBS were loaded onto one PDMS array preloaded with barcoded mRNA capture beads (ChemGenes) and settled by gravity into distinct wells. The loaded arrays were washed with PBS and sealed using a polycarbonate membrane with a pore size of 0.01 µm, which allows for exchange of buffers but retains biological molecules within each nanowell. Arrays were sealed in a dry 37°C oven for 40 min and submerged in a lysis buffer containing guanidium thiocyanate (Sigma), EDTA, 1% beta-mercaptoethanol and sarkosyl (Sigma) for 20 min at room temperature. Arrays were transferred to hybridization buffer containing NaCl (Fisher Scientific) and supplemented with 8% (v/v) polyethylene glycol (PEG, sigma) and agitated for 40 min at room temperature, mRNA capture beads with mRNA hybridized were collected from each Seq-Well array, and beads were resuspended in a master mix for reverse transcription containing Maxima H Minus Reverse Transcriptase (ThermoFisher EP0753) and buffer, dNTPs, RNase inhibitor, a 50 template switch oligonucleotide, and PEG for 30 min at room temperature, and overnight at 52°C with end-over-end rotation. Exonuclease I treatment (New England Biolabs M0293L) was used to remove excess primers. After exonuclease digestion, bead-associated cDNA denatured for 5 min in 0.2 mM NaOH with end over end rotation. Next, beads were washed with TE + 0.01% tween-20, and second strand synthesis was carried out by resuspending beads in a master mix containing Klenow Fragment (NEB), dNTPs, PEG, and the dN-SMRT oligonucleotide to enable random priming off of the beads. PCR amplification was carried out using KAPA HiFi PCR Mastermix (Kapa Biosystems KK2602) with 2.00 beads per 50 µL reaction volume. Post-whole transcriptome amplification, libraries were then pooled in sets of six (12,000 beads) and purified using Agencourt AMPure XP SPRI beads (Beckman Coulter, A63881) by a 0.6x volume ratio, followed by a 0.8x. Libraries size was analysed using an Agilent Tapestation hsD5000 kit (Agilent Genomics) with an expected peak at 1000 bp and absence of smaller primer peaks. Libraries were quantified using Qubit High-Sensitivity DNA kit and preparation kit and libraries were constructed using Nextera XT DNA tagmentation (Illumina FC-131-1096) using 800 pg of pooled cDNA library as input using index primers with format as in Gierahn et al. Amplified final libraries were purified twice with AMpure XP SPRI beads as before, with a volume ratio of 0.6x followed by 0.8x yielding library sizes with an average distribution of 650-750 pb. Libraries were pooled and sequenced together using a Illumina NovaSeq 6000 S2 Reagent kit v1.5 (100 cycles) using a paired end read structure with custom read 1 primer: read 1: 20 bases with a 12 bases cell barcode and 8 bases unique molecular identifier (UMI). Read 2: 82 bases of transcript information, index 1 and index 2: 8 bases.

### Seq-Well scRNAseq computational pipeline and analysis

Raw sequencing data were converted to demultiplexed FASTQ files using bcl2fastq2 based on Nextera N700 indices corresponding to individual arrays. Reads were then aligned to hg19 genome assembly and aligned using the Dropseq-tools pipeline on Terra (app.terra.bio). Data were normalized and scaled using Seurat R package v.3.1.0 (https://satijalab.org/seurat/), any cell with fewer than 750 UMIs or greater than 2,500 UMIs This cell-by-genes matrix was then used to create a Seurat object. Cells with any gene expressed in fewer than 5 cells were discarded from downstream analysis and any cell with at least 300 unique genes was retained. Cells with < 20% of UMIs mapping to mitochondrial genes were then removed. These objects were then merged into one object for pre-processing and cell-type identification. The combined Seurat object was log-normalized to UMI+1 and applied a scale factor of 10,000. We examined highly variable genes across all cells, yielding 2,000 variable genes. We ran the Harmony package in R (49) within the Seurat workflow to adjust for batch effects. The top 20 normalized Harmony vectors in PCA space were used as input to assessment methods. For 2D visualization and cell type clustering, we used a Uniform Manifold Approximation and Projection (UMAP) dimensionality reduction technique with “min_dist” set to 0.5 and “n_neighbors” set to 30. To identify clusters of transcriptionally similar cells, we employed unsupervised clustering as described above using the FindClusters tool within the Seurat R package with default parameters and k.param set to 10 and resolution set to 0.5.nDifferential expression analysis between the negative and positive groups of the same CD8^+^ T-cells was performed suing the Seurat package FindAllMarkers in Seurat v3 (setting ‘‘test.use’’ to bimod). For each cluster, differentially expressed (DEGs) were generated relative to all of the other cells. Gene ontology and pathway analyses from DEGs were performed using Ingenuity Pathway Analaysis (IPA) which supports statistical analysis and visualization profiles for genes and gene clusters.

### SMART-Seq2 whole transcriptome amplification and RNA sequencing

Libraries were prepared using a modified SMART-Seq2 protocol as previously reported (50, 51). In brief, based on FACS analysis (BD FACSAria™), single cells of Tetramer^+^ (HIV or CMV-specific), and tetramer^-^ cells (bulk/total CD8^+^ T-cells), were sorted into wells onto 96-well plates with lysis buffer, which contained 1 µl 10 mM dNTP mix, 1 µL 10 µM oligo dT primer, 1.9 µl 1% Triton X-100 (Sigma) plus 0.1 µl 40 U/µl RNase Inhibitor. The sealed plates were stored frozen at -80°C. Thawed plate is incubated for 3 min at 72°C and placed on ice. Next, SMART-Seq2 Whole Transcriptome Amplification (WTA) was performed: 7 µL of RT mix was added to each well and RT was carried out; then, 14 µL of PCR mix was added to each well and PCR was performed. Thereafter a cDNA clean-up was performed using 0.6x and 0.8x volumes of Agencourt AMPure XP SPRI beads (Beckman Coulter) to eliminate short fragments (less than 500 bp). Libraries were then quantified using a Qubit dsDNA HS Assay Kit (Life Technologies). Library size and quality were measured by Bioanalyzer using a High Sensitivity DNA Analysis Kit (Agilent Technologies). Sequencing libraries were prepared from WTA products using Nextera XT (Illumina). After library construction, a final AMPure XP SPRI clean-up (0.8 volumes) was conducted. Library concentration and size were measured with the KAPA Library Quantification kit (KAPA Biosystems) and a Tape Station (Agilent Technologies), respectively. Library was then constructed using Nextera XT DNA library preparation kit (Illumina FC-131-1096) using index primer. After library construction, sequences were purified using a 0.8X SPRI ratio yielding library sizes with an average distribution of 500-750 pb in length as determined using an Agilent hsD5000 Screen Tape System (Agilent Genomics). Finally, samples were sequenced on a NextSeq500 (30 bp paired-end reads) to an average depth of 5 million reads. Reads were aligned to hg19 (Gencode v21) using Top Hat (52) and estimated counts and transcripts per million (TPM) matrices generated using RSEM (53). Any samples with fewer than 5x10E5 or more than 6x10E6 aligned reads or fewer than 10,000 uniquely ex-pressed genes were removed from subsequent analysis.

### T-cell receptor sequencing

A fraction of the WTA product was further amplified with V and C primers (both α and β) for TCR amplification. Followed by a second PCR reaction incorporating individual barcodes in each well. The samples are combined, purified and sequenced using Illumina Miseq with pair-end reads. For TCR sequences, the CDR3 nucleotide sequence is extracted and translated.

### SMART-Seq2 RNA-sequencing data analysis

Cells with a minimum of 2000 genes, maximum of twenty percent mitochondrial genes were included in downstream analysis run using Scanpy version 1.4.7 (scanpy.readthedocs.io) package (54). PCA was run the log-normalized expression values of the top 1500 variable genes determined with the scanpy.preprocessing.highly_variable_genes function with inputs, (flavor=‘seurat’, batch_key=“patient”). Visualization using tSNE was run on top 10 principal components with perplexity 10 and learning rate 200. Preprocessing and clustering using Louvain clustering with resolution 0.7 were performed. Marker genes evaluated using *rank_genes_groups* function using method “t-overestem-var”. Top thirty genes were evaluated and ribosomal and mitochondrial genes were removed from gene lists. Plotting and visualization with matplotlib (55) and seaborn (56) libraries. Gene set scoring was run with the *score_genes* function with parameter “use_raw=True” using genes shared between reference gene lists and the transcriptional data. Differences in gene set score between groups were evaluated with a Kruskal Wallis test as implemented in scipy.stats.

The log of normalized MFI values for each channel associated with cells sequenced with SmartSeq2 was split into high and low bins using a Gaussian mixture model with two components using scikit-learn (57).

TCR alignment was executed using MiXCR (58). Clones represented in two or more single cells were considered to be expanded for downstream analysis. The Scirpy package was used for further downstream analysis (59).

### Quantification and statistical analysis

Graphs were plotted using Prism 9.2.0 (GraphPad Inc.) Differences between groups were analysed using Mann Whitney U-test or ANOVA multiple comparisons test (two -sided), with a p-value <0.05 considered to be statistically significant. If any other specific test is used, it has been stated in the figure legends. The values of n refer to the number of participants used in the study.

## Results

### CD8^+^ T-cells from human tonsils express a non-cytolytic T_RM_-like phenotype and are located outside the follicular GC

**Figure 1:**
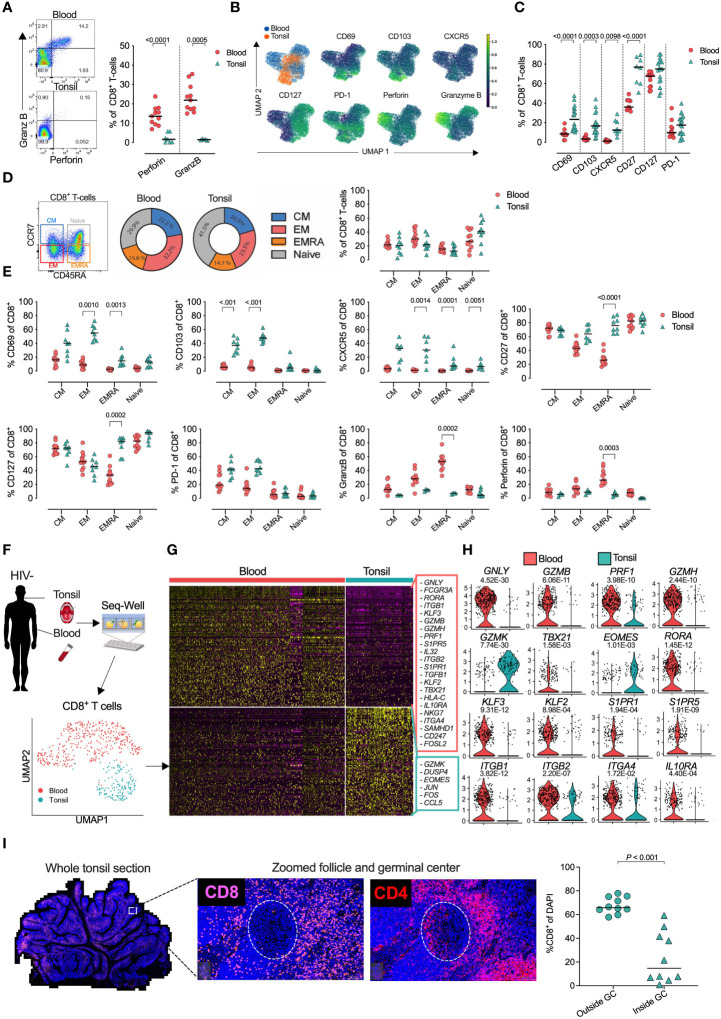
Tonsil CD8^+^ T-cell resident phenotype and transcriptional signature with distinct *in situ* location within whole tonsil section. **(A)** Blood (*n=* 11) and tonsil (*n=*18) mononuclear cells pre-gated on CD8^+^ T-cells by flow cytometry for perforin and granzyme B (GZMB). **(B)** Expression intensity of CD69, CD103, CXCR5, CD127, PD-1, Perforin and GZMB in multidimensional UMAP space for CD8^+^ T-cells, gated from total live CD45^+^ CD3^+^ T-cells. Peripheral blood (blue), tonsil tissue (orange). **(C)** Percentage of blood (*n=*11) and tonsil (*n=*7) bulk CD8^+^ T-cells expressing indicated markers. **(D)** Memory phenotype by CD45RA/CCR7 expression in blood and tonsil CD8+ T-cells. **(E)** Indicated markers compared in four CD45RA/CCR7 memory populations. **(F)** Schematic of protocol for scRNAseq of CD8^+^ T-cells from peripheral blood (*n=*3) and tonsil (*n=*2) tissue by Seq-Well S^3^ with UMAP of 496 CD8^+^ T-cells coloured by tissue source (bottom). **(G)** Heatmap of z-scored gene expression of CD8^+^ T-cells illustrating 440 differentially expressed genes between blood and tonsil with selected genes highlighted (right). **(H)** Violin plots showing selected genes for cytolytic, transcription factors, tissue-resident memory and immune suppressive markers, expressed in CD8^+^ T-cells. FDR-adjusted p<0.05; full results can be found in **Supplementary Table S1**. **(I)** Fluorescence immunohistochemistry of whole tonsil section (left) from HIV uninfected donor shown by CD8 and CD4 panels in white box zoomed area (middle) with quantification using 10 unrelated areas identified outside follicles (outside GCs) and within follicular germinal centers (inside GCs) as defined by PD-1 expression. P-values by Kruskal-Wallis corrected for multiple comparisons.

Recent studies show major phenotypic differences between CD8^+^ T-cells in circulation and those in lymphoid tissue (39, 43). To further explore these differences and examine the impact of HIV infection, we collected tonsils and peripheral blood from study participants undergoing routine tonsillectomy in clinics located within high HIV endemic areas of KwaZulu-Natal, South Africa (**Table 1**). First, we analyzed matched blood and tonsil cells from HIV uninfected participants gated on bulk CD8+ T-cells including both naïve and memory populations (**Figure S1A**). Consistent with previous work, observed reduced expression of the key T-cell cytolytic markers perforin and granzyme B (GZMB) in tonsil CD8^+^ T-cells (**Figure 1A**) (43). Overall, tonsil CD8^+^ T-cells were phenotypically distinct from the blood compartment (**Figures 1B**, **S1B**), with higher surface expression of CD69, CD103, CD27 and the follicle-homing chemokine receptor CXCR5 (**Figure 1C**), consistent with a T_RM_-like phenotype (39). While no difference in frequencies of CD45RA/CCR7 memory subsets were found between blood and tonsil CD8+ T-cells (**Figure 1D**), the differences in CD69, CD103, CXCR5, PD-1 and cytolytic markers were enriched in the non-naive CD8 T-cells (**Figure 1E**) (39, 43) and consistent with re-circulation of naïve CD8+ T-cells (60). Next, we performed single-cell RNA-sequencing (scRNAseq) on bulk CD8^+^ T-cells isolated from blood (*n=*3) and tonsils (*n=*2) using the Seq-Well S^3^ platform (47, 48) (**Figure 1F**) with similar frequencies of naïve and memory CD8+ T-cells subsets (see **Figure 1D**). We identified a distinct transcriptional programme in tonsil CD8^+^ T-cells, with 223 differentially expressed genes (DEGs) characteristic of tissue resident markers previously established for lymph nodes (61) (**Figure 1G**; **Table S1**). Canonical cytotoxic genes included *GNLY, GZMB, PRF1* and *GZMH* were enriched in blood, with the notable exception of *GZMK*, which was only expressed in tonsil CD8^+^ T-cells (**Figure 1H**). Transcription factors *TBX21, EOMES* and *RORA* also displayed compartment specific patterns, with EOMES enriched in tonsil CD8^+^ T-cells consistent with recent findings (60), and *TBX21* (T-bet) and *RORA* in blood (**Figure 1H**). *KLF2*, involved in Sphingosine-1-phosphate receptor (S1PR) expression, was associated with *S1PR1* and *S1PR5* expression, all of which were reduced in tonsil CD8^+^ T-cells, consistent with their role of sphingosine-1-phosphate-mediated egress from lymphoid tissue into circulation. In addition, tonsil CD8^+^ T-cells expressed lower levels of the integrin encoding genes *ITGB2* and *ITGA4* and an apparent complete absence of *ITGB1* consistent with a ITGB1 (CD29) associated non-cytotoxic phenotype of LN CD8^+^ T-cells under normal conditions (62) (**Figure 1H**). Thus, scRNAseq reveal a transcriptional signature of differences in blood and tonsil CD8+ T-cells (**Table S1**). When we looked for the spatial location of CD8+ T-cells in tonsils we found tonsil CD8^+^ T-cells were primarily located outside the follicular germinal centers (GCs), in contrast to CD4 T-cells found both in the cortex and within GCs where T-follicular helper cells are located (**Figure 1I**). Taken together, these data defines a distinct protein and transcription factor expression signature that is consistent with a T_RM_-like and less differentiated memory phenotype enriched in non-naïve tonsil CD8+ T-cells (31) largely confined outside GCs.

**Table 1:** Clinical characteristics of study participants (n=74).

	HIV^-^		HIV+			
	Blood (n = 21)	Tonsil (n = 20)	Blood (n = 10)		Tonsil (n = 23)	
ART status	NA	NA	ART+ (n=4)	ART- (n=6)	ART+ (n=15)	ART- (n=8)
Gender F, %	21, 100%	5, 25%	4, 100%	6, 100%	14, 93%	7, 88%
Ethnicity, B/C/I/W, n	21/0/0/0	18/2/0/0	4/0/0/0	6/0/0/0	14/1/0/0	8/0/0/0
Age (years), median (IQR)	19 (19-22)	26 (16-31)	34 (29-41)	28 (24-34)	26 (12-36)	31 (24-36)
Viral load (IQR)	NA	NA	20, (20-20)	24250 (12466-406250)	60 (20-155)	5136 (20-74111)
CD4 (cells/μL), median (IQR)	NA	NA	753	526 (388-635)	510 (222-591)	493 (484-545)

B, African/Black; C, coloured; W, White.

### PD-1 expression and T_RM_-like phenotypes are elevated in tonsil CD8^+^ T-cells from PLWH

**Figure 2:**
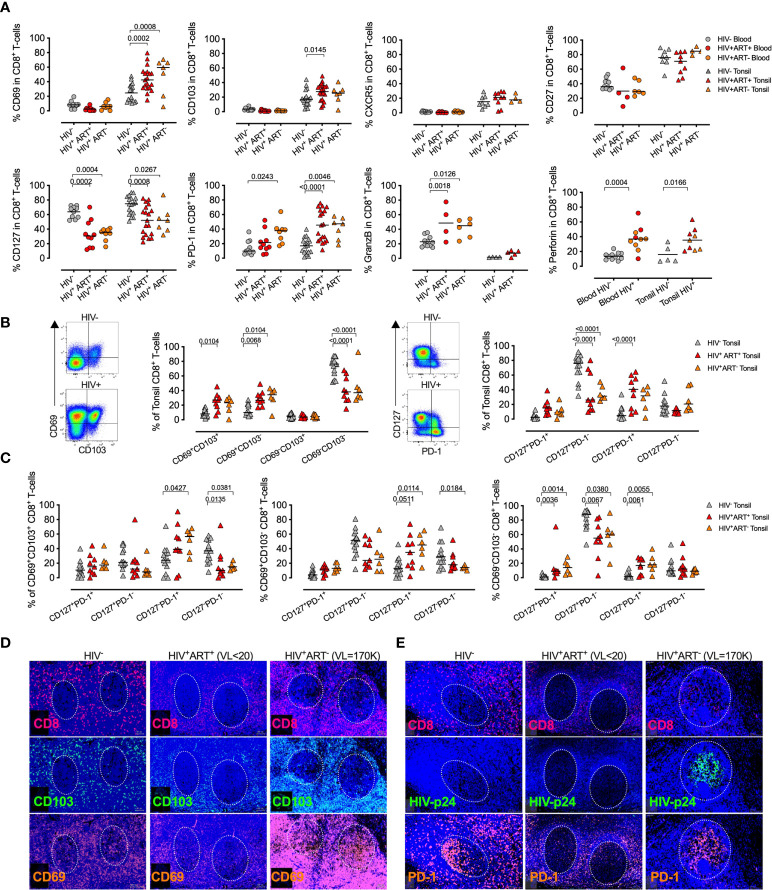
Treated HIV infection drives a tissue resident memory CD8^+^ T-cell phenotype with high PD-1 expression and distinct *in situ* tissue locations within HIV infected tonsils. **(A)** Percentage of blood (*n=*29, circles) and tonsil (*n=*44, triangles) CD8^+^ T-cells expressing CD69, CD103, CXCR5, CD27, CD127, PD-1, GZMB and perforin markers between HIV^-^ (grey), HIV^+^ART^+^ (red) and HIV^+^ART^-^ (orange). P-values were calculated using ordinary one-way ANOVA with horizontal bars representing median values with the level of significance indicated with p-value. **(B)** Representative FACS plot of CD8^+^ T-cells from HIV^-^ (grey) and HIV^+^ART^+^ (red) HIV^+^ART^-^ (orange) tonsils (*n=*29) for co-expression of CD69/CD103 and PD-1/CD127 with cumulative data shown with horizontal bars representing median values and p-values by Kruskal-wallis multiple comparisons. **(C)** CD127/PD-1 co-expression on indicated CD69/CD103 CD8+ T-cell subsets. **(D)** CD8, CD69, CD103 fluorescence immunohistochemistry of whole tonsil sections zoomed in at individual GCs for three independent donors from HIV^-^ (left), HIV^+^ART^+^ (middle) and HIV^+^ART^-^ (right) with individual markers shown and plasma viral load in brackets (above) and GCs indicated by dotted white circle. **(E)** Same as in C but for CD8, HIV-p24 and PD-1.

Next, we compared the expression of phenotypic markers in both naïve and non-naïve CD8^+^ T-cells from tonsils and blood of PLWH (**Figures S2A, B**). CD69 and CD103, involved in tissue residency, were upregulated in tonsils from PLWH, but not in matched blood from the same individuals, potentially indicating enrichment of tonsil T_RM_-like CD8^+^ T-cells after HIV infection that was not reversed by ART (**Figure 2A**). Equal CXCR5 expression in tonsil CD8+ T-cells of PLWH suggests no increased potential of GC access through the CXCR5/CXCL13 axis. Lower CD127, required for IL-7 homeostatic signaling was consistent for both blood and tonsil compartments. In contrast, PD-1 expression was highly upregulated in tonsil derived CD8^+^ T-cells of PLWH compared to HIV- irrespective of ART status, suggesting antigen specific activation (63, 64) and potentially reduced functionality (32, 65). GZMB and perforin were upregulated in both compartments in PLWH suggesting a potential increase in cytolytic activity.

Co-expression of CD69 and CD103 T_RM_ markers showed that elevated CD69 levels were most strongly associated with HIV infection, and therefore potentially increased tissue residency. Loss of CD127^+^/PD-1^-^ and expansion of CD127^-^/PD-1^+^ suggests a loss of homeostatic IL7Ra signaling and enriched exhaustive CD8+ T-cell population in tonsils from PLWH irrespective of ART mediated viral suppression (**Figure 2B**). Although these were not controlled for by naive and non-naïve cells, those differences remined significant across TRM-like (CD69^+^CD103^+/-^) and non-TRM-like (CD69^-^CD103^-^) CD8^+^ T-cell populations (**Figure 2C**).

Spatial localization of CD8+ cells in tonsils from three donors representing uninfected, viral suppressed and viremic conditions (**Figures 2D, E**) consistently showed CD8^+^ cells were located outside the GCs in the HIV negative and the ART suppressed individuals, but with low level GC infiltration in the HIV viremic donor that was consistent with increased overlapping CD103 expression in GCs from the viremic individual that also showed massive expansion of CD69 expression (**Figure 2D**). However, CD103/CD8 co-expression was also observed in the HIV uninfected donor, although to lower levels. HIV-p24 protein detection was low and restricted to non-GC areas in the viral suppressed donor, whereas the high viremic donor showed almost exclusively detection within the GCs overlapping with detection of CD8+ cells and PD-1 expression, which may indicate that CD8^+^ T_RM_-like cells can gain access to GCs with high levels of HIV-p24 detection in viremic tonsils (**Figure 2E**). Interestingly, PD-1 was also highly expressed within these GCs, but most likely reflects T-follicular helpers cells (66, 67). Thus, CD8^+^ T-cells with a T_RM_-like phenotype are increased in tonsils from PLWH and express high levels of PD-1 and perforin, but low levels of CD127, consistent with chronic activation, increased cytolytic activity and impaired homeostatic signaling that is not reversed by ART mediated viral suppression (39).

### HIV infection alters memory CD8^+^ T-cells in human tonsils

**Figure 3:**
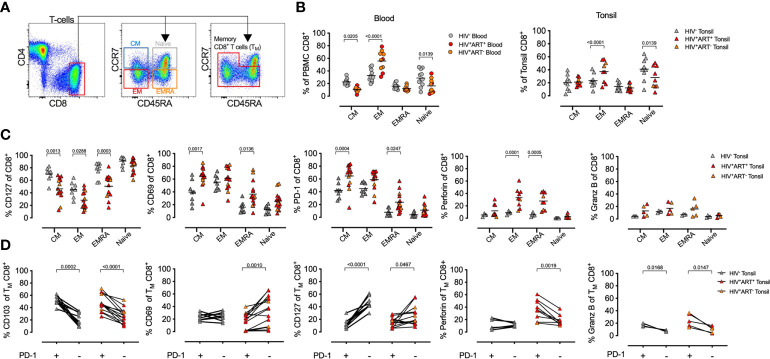
HIV infection is associated with memory CD8^+^ T-cell expansion in tonsils. **(A)** Representative FACS plot of the gating strategy of CD8^+^ T-cells from tonsil cells to detect naïve, T_N_ (CCR7^+^CD45RA^+^), central memory (T_CM_, CCR7^+^CD45RA^-^), effector memory (T_EM_, CCR7^-^CD45RA^-^) and T_EMRA_ (CCR7^-^CD45RA^+^). **(B)** Comparison of memory subset distribution within blood and tonsil CD8^+^ T-cells for central memory, T_CM_, Effector memory, T_EM_, transitional, T_EMRA_, T_Naive_ with cumulative memory subset distribution of CD8^+^ T-cells for blood (*n=*21, circles, left) and tonsil (*n=*22, triangles, right). **(C)** Distribution of tonsil central memory (T_CM_), transitional memory (T_EMRA_), effector memory (T_EM_) and naïve subsets within CD127, CD69, PD-1, perforin and GZMB expressing CD8^+^ T-cells cumulative for all study participants in HIV^-^ (grey), HIV^+^ART^+^ (red) and HIV^+^ART^-^ (orange). P-values calculated using ordinary one-way ANOVA with horizontal bars representing median values with the level of significance indicated above. **(D)** The frequency of CD103, CD69, CD127, perforin, and GZMB cells measured on PD-1^+^ (left) and PD-1^-^ (right) CD8^+^ T-cells from tonsil in HIV+ (red) and HIV^-^ (grey) individuals. P-values calculated using Paired Student’s t-test. Horizontal bars represent median values.

Expression of inhibitory receptors, such as PD-1, is linked to T-cell differentiation (68) and reduced functionality in the blood of PLWH (32, 37), but the role of PD-1 expression on CD8^+^ T-cells in lymph nodes remains less clear. To examine this further, we first identified CD8^+^ T-cell memory subsets by CD45RA and CCR7 expression (69) (**Figure 3A**), and found that HIV infection enriches for effector memory cells with reduced naïve CD8^+^ T-cells in both blood and tonsil (**Figure 3B**). The effect of HIV on CD127, PD-1 and perforin in both tonsils and blood was confined to memory populations with increased expression of CD69 on T_CM_ and T_EMRA_ in tonsils of PLWH (**Figure 3C**) consistent with recent data from HIV infected lymph nodes (39). CD103 expression, however, was unchanged across all memory subsets, suggesting the upregulation of this T_RM_ marker in PLWH was not driven by specific T-cell activation (**Figure S3A**).

Next, we focused on the enhanced PD-1 expression in PLWH and gated on memory subsets alone (CD8^+^ T_M_-cells) in blood and tissue and determined the co-expression of the remaining phenotypic markers (**Figure 3D**). We found PD-1^+^ CD8^+^ T_M_-cells in tonsils were generally CD103^+^, irrespective of HIV status, but with lower CD69 expression on PD-1^+^ CD8^+^ T_M_-cells in PLWH. Perforin and GZMB expressing CD8^+^ T_M_-cells in PLWH were generally PD-1^+^ in PLWH. Collectively, these data show that markers associated with tissue residency, immune activation and cytolytic activity are driven by memory subsets and altered by HIV infection.

### HIV associated phenotype changes are enriched in HIV-specific tonsil CD8^+^ T-cells

**Figure 4:**
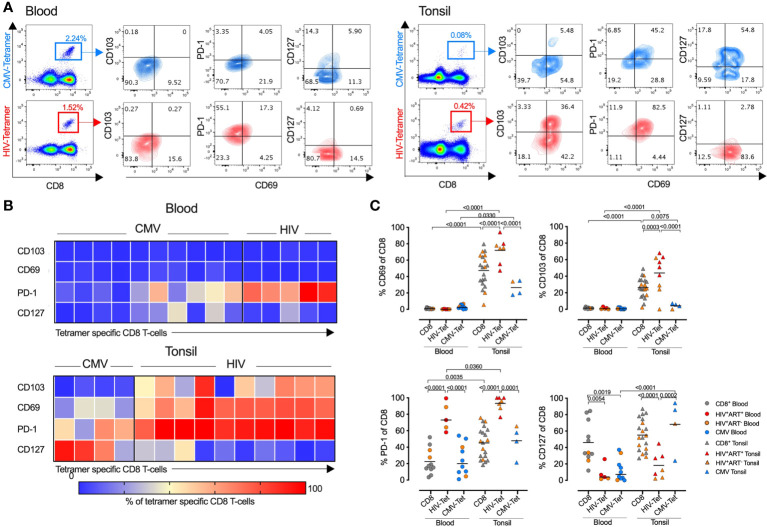
HIV specific CD8^+^ T-cells express high levels of PD-1 and CD69 compared to CMV- and non-specific CD8^+^ T-cells in tonsils. **(A)** Representative FACS plots showing CMV- (top) and HIV-specific (bottom) tetramer stains of blood (left) and tonsil tissue (right) from the same HIV^+^ individual with indicated markers listed. **(B)** Heatmap showing expression frequencies for the indicated markers among CD8^+^ T-cells from CMV tetramer and HIV tetramer specific gated CD8^+^ T-cells in blood (top) and tonsil (bottom) with frequencies for each tetramer population indicated by the intensity bar below from blue (0%) to red (100%). **(C)** Frequencies of CD69, CD103, PD-1 and CD127 from HIV-, CMV-, and non-specific (‘CD8’) CD8^+^ T-cells within blood (*n=*11, left) and tonsil (*n=*20, right) matched tissue. P-values calculated using ordinary one-way ANOVA with horizontal bars representing median values with the level of significance indicated above.

We next explored the role of antigen specificity and used HLA-class I tetramers to detect HIV-specific, cytomegalovirus (CMV)-specific and non-HIV/CMV specific (bulk) CD8^+^ T-cells in donor matched tonsil and blood (**Figure 4A**). Consistent with bulk CD8^+^ T-cells, CD69 and CD103 expression were absent on both HIV and CMV specific T-cells in the blood, whereas in tonsils the majority of HIV specific CD8^+^ T-cells expressed both CD69 and CD103 consistent with a T_RM_-like phenotype. In contrast, although CMV specific CD8^+^ T-cells expressed CD69 more frequently in tonsils compared to blood, this was lower than both HIV-specific and bulk CD8^+^ T-cells, and these cells almost entirely lacked CD103 expression (**Figures 4B, C**). PD-1 expression was significantly more frequent on HIV-specific CD8^+^ T-cells compared to CMV specific cells and higher in tonsils compared to blood suggesting more cognate antigen stimulation (37, 38) and consistent with lower CD127 expression and terminally differentiated HIV specific CD8^+^ T-cells in tonsils (**Figures 4B, C**). Thus, HIV-specific CD8^+^ T-cells in HIV-infected tonsils showed enriched TRM-like expansion with increased PD-1 and reduced CD127 expression compared to bulk and CMV specific CD8^+^ T-cells suggesting these changes are, at least in part, driven by HIV antigenic stimulation.

### Single-cell transcriptional profiling identifies distinct subsets within heterogenous HIV specific tonsil CD8^+^ T_RM_-cells

**Figure 5:**
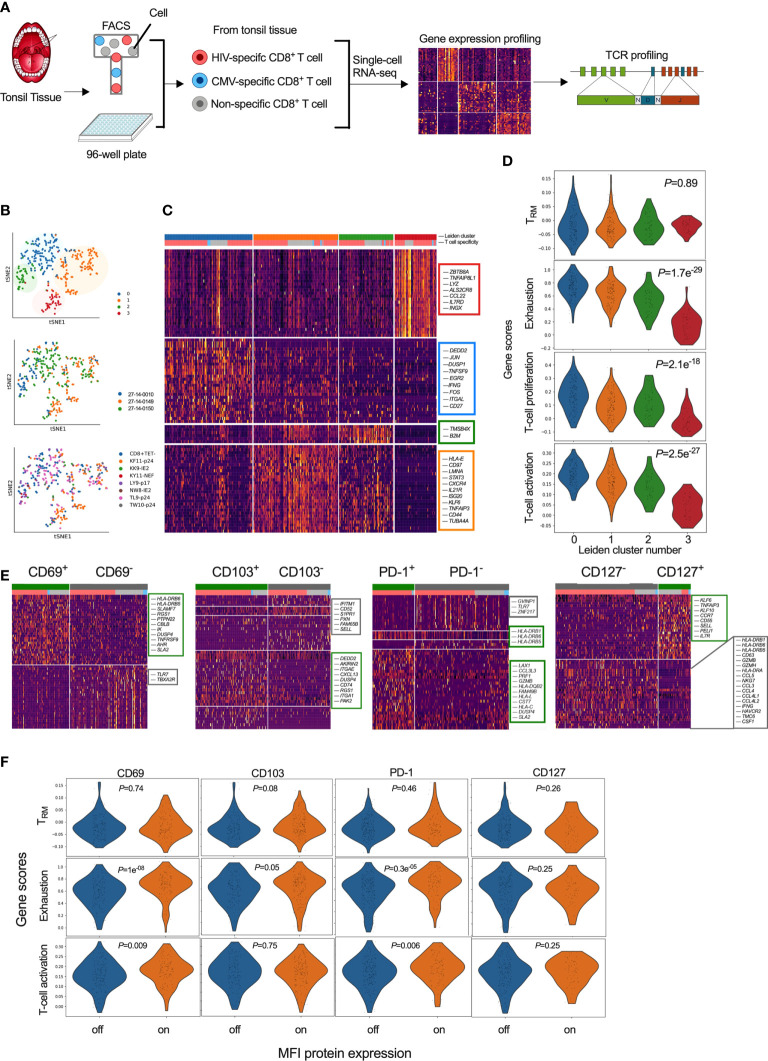
Single-cell transcriptional profiling of CD8^+^ T-cells within HIV infected tonsils reveals heterogenous and distinct subsets grouped by T-cell exhaustion gene set enrichment scores. **(A)** Workflow of scRNAseq from HIV infected tonsil isolated CD8^+^ T-cells pre-sorted on HIV-, CMV- and ‘non-specific’ CD8^+^ T-cells from HIV infected participants (*n=*3) (see **Supplemental Table S2**). **(B)** Dimensionality reduction using tSNE on scRNAseq cells coloured by Louvain cluster (top), participant ID (middle), and tetramer specificity (bottom). **(C)** Heatmap of z-scored gene expression of top differentially expressed genes (t-test) between Louvain clusters from scRNAseq data with cells grouped by Louvain cluster, genes grouped by hierarchical clustering (full gene lists in **Supplemental Table S3**). **(D)** Gene set enrichment scores for each of the 4 Louvain clusters (0-3) shown for ‘T_RM_’ (39), ‘Exhaustion’ (70), ‘proliferation’ and ‘activation’ published gene lists (71, 72). **(E)** Heatmaps of z-scored gene expression of top differentially expressed genes (t-test) between single cells with high and low normalized MFI values of CD69, CD103, PD-1 and CD127. Selected genes labelled in plot, full gene lists in **Supplemental Tables S4, S5**. **(F)** Gene lists from **(E)** scored against the published gene lists as indicated in D.

To further characterise HIV specific CD8^+^ T-cells in tonsils, we applied an optimized SMART-seq2 protocol to perform scRNAseq on single-cell tetramer sorted HIV- and CMV-specific CD8^+^ T-cells as well as bulk CD8^+^ T-cells (non-specific CD8^+^ Tet^-^), from tonsils of participants with chronic untreated HIV infection (**Table S2**; **Figure 5A**). We first created a cells-by-genes expression matrix, performed variable gene selection and unsupervised clustering using graph-based clustering and identified four distinct CD8^+^ T-cell clusters (**Figure 5B**). Although the relative frequency in each cluster varied between donors, we found cells from each donor represented in all of the 4 clusters. Furthermore, all of the 7 different tetramer specific and non-specific CD8^+^ T-cells were represented in each cluster, suggesting they describe some differences in underlying cellular states rather than donor and/or antigen specific effects, although that cannot be completely excluded. Differential gene expression analysis among these four clusters revealed notable molecular differences (**Figure 5C** and **Table S3**). Genes in cluster 0 (blue) included *JUN, JUNB, FOS, FOSB* and *ATF*, which together form the AP-1 transcription factor, and the MAPK signaling genes *DUSP1 and DUSP2*, both of which control cellular differentiation and proliferation during viral infection (73) along with *EGR2*, which is also expressed by cluster 0 (74, 75). Together with the expression of *TNFSF9*, involved in lymphocyte activation and *IFNG*, this transcriptional signature suggests that cells in cluster 0 are highly activated and proliferating. Cluster 1 (orange) marks genes involved in activation – *HLA-E*, *CFL1, STAT3, CD97, LMNA* and along with the transcription factor KLF6. Cluster 2 contains mostly ribosomal genes and potentially indicates increased activity of protein machinery, as observed in the lymph nodes of elite controllers (44). Cluster 3 contains *IL17RD*, involved in the T_H_17 response, and *CCL22*, interacting with CCR4, along with the TNF-α induced protein 8 encoded by *TNFAIP8L1* and *AXL* involved in tyrosine kinase signaling (**Figure 5C**).

To ascribe functional states to these 4 gene clusters (‘Leiden clusters 0-3’), we scored them against gene sets derived from: i) tissue resident memory CD8^+^ T-cells (T_RM_) in human lymph nodes (39) (**Table S2**); ii) exhausted T-cells (70) (**Table S13**); and, iii) T-cell proliferation and iv) T-cell activation (71, 72). We found no difference in T_RM_ scores (P=0.89), but a strong difference in exhaustion, proliferation and activation profiles (P<0.0001), which were highest for cluster 0 followed by cluster 1 and 2. Cluster 3 scored low in all gene scores, except T_RM_, indicating more quiescent cellular states despite the majority of these cells being HIV specific and expressing a T_RM_-like phenotype (**Figure 5D**). Thus, single cell transcriptional resolution reveals heterogeneity between HIV-specific CD8^+^ T-cells with respect to exhaustion, activation and proliferation while the similar T_RM_ gene set enrichment score is consistent with these cells coming from tonsils.

### CD69 and PD-1 expression marks transcriptionally fates of immune exhaustion and activation signatures on HIV specific tonsil CD8^+^ T-cells

To determine if surface marker expression could predict cellular transcriptional state, we first compared gene expression levels (mRNA) with matched surface protein expression (MFI) HIV, CMV and non-CMV/HIV specific single CD8^+^ T-cells that was consistent with higher protein expression of CD69, CD103 and PD-1, and lower CD127 expression on HIV specific compared to CMV-specific CD8^+^ T-cells (**Figure S4A**). Direct correlation between mRNA and MFI abundance was found only for *IL7R* and CD127 (**Figure S4B**). To test whether differences in protein expression of these markers were linked to specific gene signatures, despite inconsistent detection of the genes themselves, we applied MFI clustering based on our protein index markers to generate ‘on-off’ signatures for each protein marker (**Figure S4C**) and used that to directly compare genes associated with protein expression (**Figure 5E**). We found CD69 protein surface expression to be associated with *HLA-DRB6, HLA-DRB5, SLAMF7* and *TNFRSF9* (4-1BB/CD137), known to regulate CD8^+^ T-cell clonal expansion and associated with recent TCR-triggered activation (76) (**Table S4**), CD103^+^ cells were associated with CD8^+^ T_RM_ integrin markers (*ITGA1, ITGAE*), chemokine receptors *(CXCL13*, *RGS1*) and G-protein-signaling genes involved in modulating T-cell trafficking (**Table S5**) (77). In contrast, CD103^-^ cells expressed genes associated with tissue egress (*S1PR1)* and the associated transcription factor *KLF2* along with *SELL* (CD62L), suggesting that these cells can re-circulate *via* lymph and peripheral blood (39, 60, 78). PD-1^+^ cells were almost exclusively HIV specific and expressed genes linked to cytotoxicity (*PRF1, GZMB, CCL3L3, CST7)* and activation (*HLA-DR, HLA-DQ, HLA-L, HLA-C*) (**Table S6**) while CD127^+^ were mostly non-specific and expressed genes associated with naïve like cells (*CCR7, SELL*, *IL7R)* in contrast to CD127^-^ cells associated with chemokine ligands (*CCL4L2, CCL4L1, CCL3, CCL4, CCL5*) and IL-12-induced effector molecules (*IFNG, GZMH, GZMB, NKG7*) and T-cell activation (*HLA-DRA, HLA-DRB1, HLA-DRB6, HLA-DRB5, CD63*) (**Table S7**).

Next, we scored these cells determined by differential protein expression against the T_RM_, exhaustion and T-cell activation gene lists used above (see **Figure 5D**). No significant differences in their T_RM_ scores were observed for any of the proteins measured (**Figure 5F**). In contrast, gene set enrichment scores associated with exhaustion and T-cell activation was higher in CD69^+^ and PD-1^+^ cells (P<0.0001) (**Figure 5F**). These data show that the HIV specific CD8^+^ T_RM_-like population in tonsils is heterogenous and that CD69 and PD-1 surface expression are enriched for transcriptional signatures of exhaustion and activation, but not necessarily tissue residency.

### HIV specific tonsil CD8^+^ T-cells from a natural HIV controller are linked to low immune activation and non-cytolytic gene signatures compared to uncontrolled HIV infection

**Figure 6:**
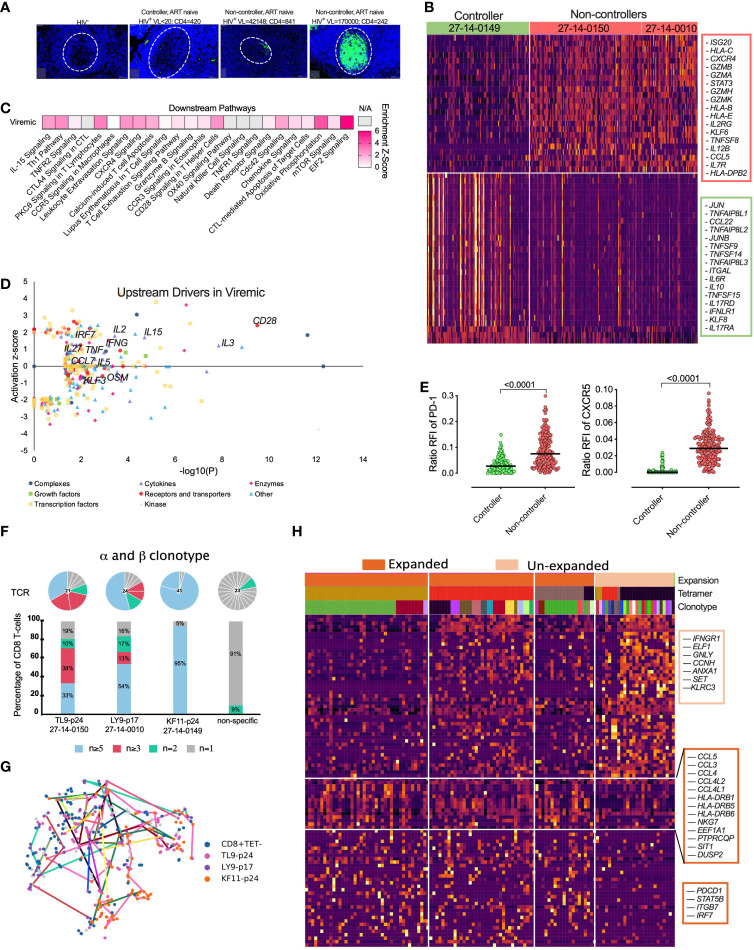
Natural HIV ‘controllers’ display reduced HIV specific CD8^+^ T-cell cytolytic transcriptional signatures and PD-1 expression compared to viremic ‘non-controllers’ linked to expanded TCR clonotypes. **(A)** Fluorescence immunohistochemistry of HIV-24 protein (green) for 4 donors of HIV^-^, HIV^+^ ‘controller’ and two HIV^+^ART^-^ ‘non-controllers’ with plasma viral load and absolute blood CD4 counts listed above. **(B)** Heatmap showing differentially expression of 300 featured genes with selectively specifically expressed genes marked (right) among 1,622 differentially genes (**Supplemental Table S12**). **(C)** Selected canonical pathways by Ingenuity Pathway Analyzer (IPA). **(D)** Upstream drivers of pathways significant by IPA of DEGs. For directionally annotated pathways, a *Z*-score is calculated to represent up- or downregulation of the driver or pathway. If a driver or pathway is not directionally annotated in IPA, or there are not enough genes in the list to calculate a *Z*-score, N/A is reported. See **Supplemental Table S9** for the full IPA results. **(E)** Relative fluorescence intensity (RFI) for PD-1 and CXCR5 expression for each HIV specific CD8^+^ T-cell comparing ‘controller’ and ‘non-controller’ participants. **(F)** The TCR distribution of KF11-p24, TL9-p24 and LY9-p17 tetramer specific CD8+ T-cells from 27-14-0149, 27-14-0150 and 27-14-0010, respectively (see **Supplemental Table S2**) with unique (n = 1), double (n = 2), triplet (n ≥ 3), and expanded (n ≥ 5) with bars colored in grey, green, pink and blue representing the fraction of cells belonging to groups of clonotypes with either 1, 2, 3-4 or more than five clonotypes, respectively. Pie chart above each bar illustrates the composition of every individual alpha-beta-TCR. **(G)** tSNE projection of scRNAseq profiled flow-sorted CD8^+^ T-cells on the lovain clusters (see **Figure 5B**) with colored lines connecting cells sharing the same CDR3 sequence. **(H)** Heatmap of z-scored gene expression of top differentially expressed genes (t-test) between Louvain clusters from scRNAseq data. Cells grouped by expansion of clonotype, genes grouped by hierarchical clustering. Full gene lists in **Supplemental Table S10**.

One of our HIV infected tissue donors maintained undetectable plasma viral load in the absence of ARV drugs in plasma by mass spectrometry (see **Table S2**) that is consistent with expression of protective HLA class I alleles and presentation of KF11-p24 and TW10-p24 CD8^+^ epitopes associated with viral control (21, 79, 80), hereafter referred to as “controller”. First, we confirmed the low level of antigenemia in the controller by immunohistochemistry and observed extremely low levels of HIV-p24 within the GCs compared to tonsils from viremic donors highlighting the GCs as HIV sanctuary sites (**Figure 6A**). We used this opportunity to determine the signatures of HIV-specific CD8^+^ T-cells from tonsils in settings of low HIV antigen in the absence of ART and performed a direct comparison of scRNAseq profiles of HIV specific CD8^+^ T-cells between controller (*n* = 1) and non-controllers (*n = 2*) (**Figure 6B**). We identified genes associated with cytolytic activity (*GZMA, GZMB, GZMH*, *GZMK)*, interferon stimulating gene 20 (*ISG20*) and downstream *STAT3* signalling in HIV specific CD8^+^ T-cells in non-controllers compared to controller (**Figure 6B**, **Table S12**). In contrast, CD8^+^ T-cells from the controller expressed the AP-1 transcription factor complex controlling cellular differentiation *TNFSF9, TNFS14, TNFS15* and *JUN, JUNB* (**Figure 6B**) consistent with lymph node CD8^+^ T-cells from elite controllers (44). Pathways analysis of 1,622 significantly DEGs revealed multiple distinct functional differences including elevated pathways of IL-15 signaling, Th1 pathways, extravasation, T-cell exhaustion, GZMB and increased metabolic activity, such as oxidative phosphorylation, mTOR and EIF2 signalling (**Figure 6C**; **Table S13**). Upstream driver analysis predicted activating cytokines involved in type I T-cell responses (IL-15, IL-2, IFN-γ and TNF-α) (**Figure 6D**; **Table S9**). Although these data are consistent with low expression of PD-1 and CXCR5 in controller HIV specific CD8^+^ T-cells (**Figure 6E**), only limited donors (n=3) were available for this analysis.

Next, we determined the clonal distribution and found clear α-β matched clonotype expansion of HIV specific CD8^+^ T-cells (**Figure 6F**), (**Figure S5B, C**). We linked each HIV-specific CD8+ T-cell and paired α-β TCR clonotype to its matched transcriptome (see **Figure 5B**) and found that expanded clonotypes were represented across 2 or more of the different transcriptional clusters (**Figure 6G**) identified previously (see **Figure 5B**), indicating that the TCR-antigen interaction does not determine transcriptional state *per se*. However, gene expression analysis between enriched vs unenriched α-β clonotypes revealed a signature of activation (*HLA-DRB1/DRB5/DRB6*) and chemokine-ligands (*CCL3/4/5, CCL4L1/2*) involved in T-cell trafficking and homing (**Figure 6H**). This gene signature was enriched for *PDCD1* (PD-1) and consistent with the hypothesis that PD-1 expression is driven by T-cell activation and expansion. Expanded clonotypes also expressed higher levels of integrin *ITGB7* and *IRF7* involved in type I interferon induction in lymphoid tissue and *STAT5B* TCR signaling. Consistent with these data, unexpanded cells, defined by unique clonotypes, expressed lower levels of T-cell activation genes (**Figures 6H**, **S5D**; **Tables S9-S11**).

These data support the hypothesis that the activated and exhausted phenotypes observed in HIV-specific CD8^+^ T-cells in tonsils are driven by antigen specific activation, or its inflammatory sequelae because they are reduced in the absence of antigenic stimulation in controllers. Consistently, expanded clonotype transcriptomes are also associated with signatures of activation, trafficking and exhaustion. Our data from limited donors support the potential importance of non-cytolytic CD8^+^ T-cell activity in controlling viral replication within lymphoid tissues (44).

## Discussion

Over recent years, in-depth studies have advanced our understanding of T-cells in human organs, in particular the phenotype differences between peripheral blood CD8^+^ T-cells and CD8^+^ T_RM_ cells (81, 82). The past decade has also established that a major HIV viral reservoir, responsible for viral rebound after cessation of antiretroviral treatment, is located in mucosal and lymphoid tissue sites (83–85). However, little is known about antiviral HIV specific CD8^+^ T-cells located in close proximity to HIV infected cells in these tissue sites, which is important if they are to be leveraged to help purge the HIV reservoir.

In this study, we made an in depth analysis of CD8^+^ T_RM_-like cells in HIV infected tonsils by applying transcriptional, clonotype and phenotypic characterisation of bulk and HIV specific CD8^+^ T-cells combined with *in situ* HIV-p24 and immune marker detections. We found that HIV infection has a dramatic impact on the expression of CD8^+^ T_RM_ markers in tonsils and that the majority of HIV-specific CD8^+^ T-cells express high levels of PD-1, CD69 and CD103 and low levels of CD127. These differences were much greater in HIV specific CD8^+^ T-cells compared to either CMV specific or bulk CD8^+^ T-cells in the same tonsils. Our single-cell transcriptional profiling revealed subclustering of CD8^+^ T-cells in tonsils and indicates that the upregulation of CD69, a marker for both T_RM_ and activation, and PD-1 on these cells is linked to transcriptional signatures of activation, differentiation, interferon stimulating genes and exhaustion. Moreover, CD8^+^ T-cell ancestry analysis suggests that these exhaustion signatures are enriched in expanded HIV-specific clonotypes, suggestive of ongoing proliferation. Finally, PD-1 and CD69 expression were lower on HIV-specific CD8^+^ T-cells isolated from the tonsils of a natural controller that also lacked the signatures of activation, immune exhaustion and displayed enhanced levels of non-cytolytic T-cell genes, in contrast to non-controllers.

Our findings also indicate that the subset composition and phenotype of peripheral blood CD8^+^ T-cells do not reflect that of tonsil tissue, highlighting the need to study relevant tissue sites. Single-cell transcriptional profiling comparing tonsil and blood defined core CD8^+^ T_RM_ signatures characterised by lower cytolytic programs, whereas blood CD8^+^ T-cells showed consistent re-circulating profiles. This is in line with recently published data showing that cytolytic CD8^+^ T-cells are restricted to the intravascular circulation (60), and questions the relationship between T-cell functionality measured in the blood and what manifests within lymphoid structures, such as the tonsil.

Both CD8^+^ T-cells from blood and tonsil showed a predominantly memory phenotype that in tonsil tissue were enriched for CD69, which is consistent with expression of transcription factors involved in preventing tissue egress. We found increased CD69 expression on CD8^+^ T_EM_ from HIV infected tonsils that were almost double that of CD8^+^ T_EM_ cells from HIV uninfected tonsils and consistent with observations in lymph nodes (39). In contrast to conventional lymph nodes, palatine tonsils belong to the oral mucosal tissue and possess crypts and an epithelial barrier expressing E-cadherin, a ligand for CD103 involved in tissue retention of CD8^+^ T_RM_ (14, 18), which we found upregulated by HIV infection and co-expressed with CD69. Interestingly, approximately a quarter of the CD69 expressing CD8^+^ T-cell population in HIV infected tonsils were CD103^+^, which is distinct from that found in LNs and highlights the existence of compartment specific differences between these different secondary lymphoid organs (39). The upregulation of CD69 and CD103 may suggest that HIV infection increases the retention of CD8^+^ T_RM_-like cells in tonsils, which could be well placed to participate in a durable response to ongoing viral replication in lymphoid tissue.

Elevated CD8^+^ T_RM_ marker expression is also consistent with our observations of strong upregulation of both PD-1 and cytolytic markers on bulk and memory CD8^+^ T-cells. Thus, the increased frequency of CD8^+^ T_RM_-like cells in HIV-infected tonsils suggests that these cells are continuously engaged in antiviral activities at the site of the viral reservoir. Interestingly, we found the majority of CD8^+^ T-cells located outside the GCs, but that GCs contained the vast majority of the HIV reservoir suggesting that CD8^+^ T-cells fail to accumulate in large numbers in B-cell follicles (42) and add to the limited knowledge of the location of HIV-specific CD8^+^ T-cells *in situ* (40).

When we performed a peptide-MHC tetramer-driven analysis of antigen specific CD8^+^ T-cells, we consistently found increased expression of T_RM_ markers and PD-1 expression on HIV specific compared to CMV specific CD8^+^ T-cells in tonsils. In particular, the HIV specific CD8^+^ T-cells were enriched in the distinct T_RM_ and PD-1^high^/CD127^low^ phenotype compared to the matched bulk CD8^+^ T-cells and further support that these cells are retained in HIV infected tonsils and potentially involved in the antiviral response. Comparisons to CD8+ T-cells specific for non-chronic viral infections, such as FLU and SARS-CoV-2 specific cells would help to establish if these changes are driven by continuous antigen exposure. Moreover, unsupervised clustering of HIV specific T_RM_-like cells by single-cell transcriptomes revealed that distinct subsets related to exhaustion, activation and proliferation exist. We also found distinct transcriptional gene sets by cell surface marker separation alone. For example, CD69 and PD-1 expression appeared to be associated with gene sets of exhaustion and activation in contrast to CD127 expression that was enriched in the non-HIV specific cells and consistent with a naïve-like and re-circulating transcriptional profile (60). This is also consistent with the *SELL, KLF2 and S1PR1* transcripts within the CD103^-^ CD8^+^ T-cells. Thus, re-circulating signatures were enriched in the CD127^+^ and CD103^-^ CD8^+^ T-cells that also contains the majority of non-HIV specific CD8^+^ T-cells in this analysis and further support that HIV specific CD8^+^ T-cells are tissue resident (39, 44).

The inclusion of tonsil tissue from a natural HIV controller allowed us to compare the HIV specific CD8^+^ T-cells to those purified from viremic ART naïve individuals. Although our study is limited to only one rare HIV controller with available lymphoid tissue, this is to our knowledge the second observation in human tissue (44) and the first in tonsils that warrants further investigation. We found distinct signatures showing that CD8^+^ T_RM_-like cells within HIV ‘controlled’ tonsils lacked transcripts encoding cytolytic effector molecules, such as *GZMA, GZMB, GZMH* and *GZMK*, while tonsil CD8^+^ T-cells from viremic individuals was characterized by upregulated pathways involved in IL-15 signaling, GZMB signaling, T-cell apoptosis/exhaustion and oxidative phosphorylation. Thus, the active metabolisms in these cells support our hypothesis above that increased antigen load in viremic compared to HIV controlled tonsils is linked to CD8^+^ T-cell activation and exhaustion. Although the cause and effect of low antigen levels and HIV control in lymphoid tissue remain unknown, the lower cytolytic profile from HIV controlled tonsil CD8^+^ T-cells is consistent with a recent study in HIV infected lymph nodes from elite controllers and further support the hypothesis of non-cytolytic features of CD8^+^ T_RM_ in elite controllers (44). Whether HIV specific CD8^+^ T-cells within lymphoid tissue from viremic donors can be reverted into low exhaustive and low cytolytic elite-like controller function by PD-1/PD-L1 inhibition (86, 87) remains to be seen (88). However, elite control may not solely be explained by their cellular immune responses, but also linked to distinct host genome viral integration sites (89).

In summary, we show that HIV infection enriches for a CD8^+^ T_RM_-like phenotype located outside the GCs in human tonsils collected from PLWH in HIV endemic areas in South Africa and that the HIV specific tonsil CD8^+^ T-cells match a non-vascular transcriptional programme (60) with high levels of PD-1 and exhaustive transcriptional signatures linked to expanded clonotypes. This, combined with low PD-1 and cytolytic profiles in our viral ‘controller’, suggests that immunotherapy should be targeted to include CD8^+^ T_RM_ cells relevant for HIV cure or remission strategies recently explored in animal models (90, 91).

## Data availability statement

The scRNA-Seq count data presented in this study are available in the NCBI GEO repository, accession number GSE215127.

## Ethics statement

The studies involving human participants were reviewed and approved by Biomedical Research Ethics Committee (BREC) BE061/13 of the University of Kwazulu-Natal in Durban, South Africa. The patients/participants provided their written informed consent to participate in this study.

## Author contributions

RF performed experiments and prepared the manuscript. SN and RF performed transcriptional analysis. OA, SWK, AS, AN, and JG contributed to experimental work. NM, DR and FK coordinated human sample collection. SB contributed with reagents. FA, JZP, ALS, RB, KM, WK contributed surgical human tissue samples. TN and PG contributed samples. SN, TO, TN, PG provided intellectual input. AKS and BB supervised data analysis and provided intellectual input. HK conceptualized and supervised the work with intellectual input from SN, AKS and AL. All authors contributed to the article and approved the submitted version.

## Funding

HK is supported by the Wellcome Trust (202485/Z/16/Z). AL is supported by the Wellcome Trust (210662/Z/18/Z). This work was supported through the Sub-Saharan African Network for TB/HIV Research Excellence (SANTHE), a DELTAS Africa Initiative (DEL-15-006). The DELTAS Africa Initiative is an independent funding scheme of the African Academy of Sciences (AAS) Alliance for Accelerating Excellence in Science in Africa and supported by the New Partnership for Africa’s Development Planning and Coordinating Agency (NEPAD Agency) with funding from the Wellcome Trust (107752/Z/15/Z) and the United Kingdom government. HNK and AS were supported by SANTHE. AKS was supported, in part, by the Searle Scholars Program, the Beckman Young Investigator Program, the NIH (5U24AI118672), a Sloan Fellowship in Chemistry, and the Bill and Melinda Gates Foundation. This research was funded in part by the Africa Health Research Institute through its Strategic Core Award by the Wellcome Trust (201433/A/16/A). This publication was supported by a Subagreement from the Johns Hopkins University with funds provided by Grant No. UM1AI164566 from the National Institute of Allergy and Infectious Diseases.

## Acknowledgments

We wish to thank the participants for their contribution to this study, the staff at the Africa Health Research Institute (AHRI) and associated medical and hospital clinical staff at Stanger and Addington Hospital in KZN. Its contents are solely the responsibility of the authors and do not necessarily represent the official views of the National Institute of Allergy and Infectious Diseases or the Johns Hopkins University. The views expressed in this publication are those of the authors and not necessarily those of AAS, NEPAD Agency, Wellcome Trust, or the United Kingdom government. For the purpose of open access, the author has applied a CC BY public copyright license to any Author Accepted Manuscript version arising from this submission.

## Conflict of interest

AKS reports compensation for consulting and/or SAB membership from Merck, Honeycomb Biotechnologies, Clarity, Repertoire Immune Medicines, Ochre Bio, Third Rock Ventures, Hovione, Relation Therapeutics, FL82, Empress Therapeutics, and Dahlia Biosciences.

The remaining authors declare that the research was conducted in the absence of any commercial or financial relationships that could be construed as a potential conflict of interest.

## Publisher’s note

All claims expressed in this article are solely those of the authors and do not necessarily represent those of their affiliated organizations, or those of the publisher, the editors and the reviewers. Any product that may be evaluated in this article, or claim that may be made by its manufacturer, is not guaranteed or endorsed by the publisher.
