# Supplementary material for: HIV specific CD8+ TRM-like cells in tonsils express exhaustive signatures in the absence of natural HIV control
Source: Front Immunol. 2022 Oct 18;13:912038. doi: 10.3389/fimmu.2022.912038 (PMC9623418; doi:10.3389/fimmu.2022.912038)
Supplement: Supplementary file 10 [file DataSheet_1.docx]

Supplementary Material

**Supplementary information**

Supplementary information includes 5 figures (Figure S1-S5) and 14 tables (Table S1-S14).

**Supplementary Tables**

**Table S1.** List of differentially expressed genes of HIV^+^ CD8^+^  T cells between Blood and Tonsil.

**Table S2.** The patient information and the responses from each patient sequenced.

**Table S3.**  List of differentially expressed genes between of HIV and CMV-specific CD8^+^ T cells from scRNA-seq .

**Table S4.**  List of differentially expressed genes between CD8^+^ CD69^+^ and CD8^+^ CD69^-^

**Table S5.**  List of differentially expressed genes between CD8^+^ CD103^+^ and CD8^+^ CD103^-^

**Table S6.**  List of differentially expressed genes between CD8^+^ CD127^+^ and CD8^+^ CD127^-^

**Table S7.**  List of differentially expressed genes between CD8^+^ PD-1^+^ and CD8^+^ PD-1^-^

**Table S8.**  List of significant genes up-/downregulated between Viremic and Controller

**Table S9.**  Gene Set Analysis Results Using Ingenuity Pathway Analysis

**Table S10-S12.** List of differentially expressed genes between clonotype expanded and unexpanded.

**Table S13.** TCRα and TCRβ chain reconstruction from full-length single-cell transcriptome of every single cell.

**Table S14.** Resource Table

**Supplementary figure 1 related to Figure 1.**

**
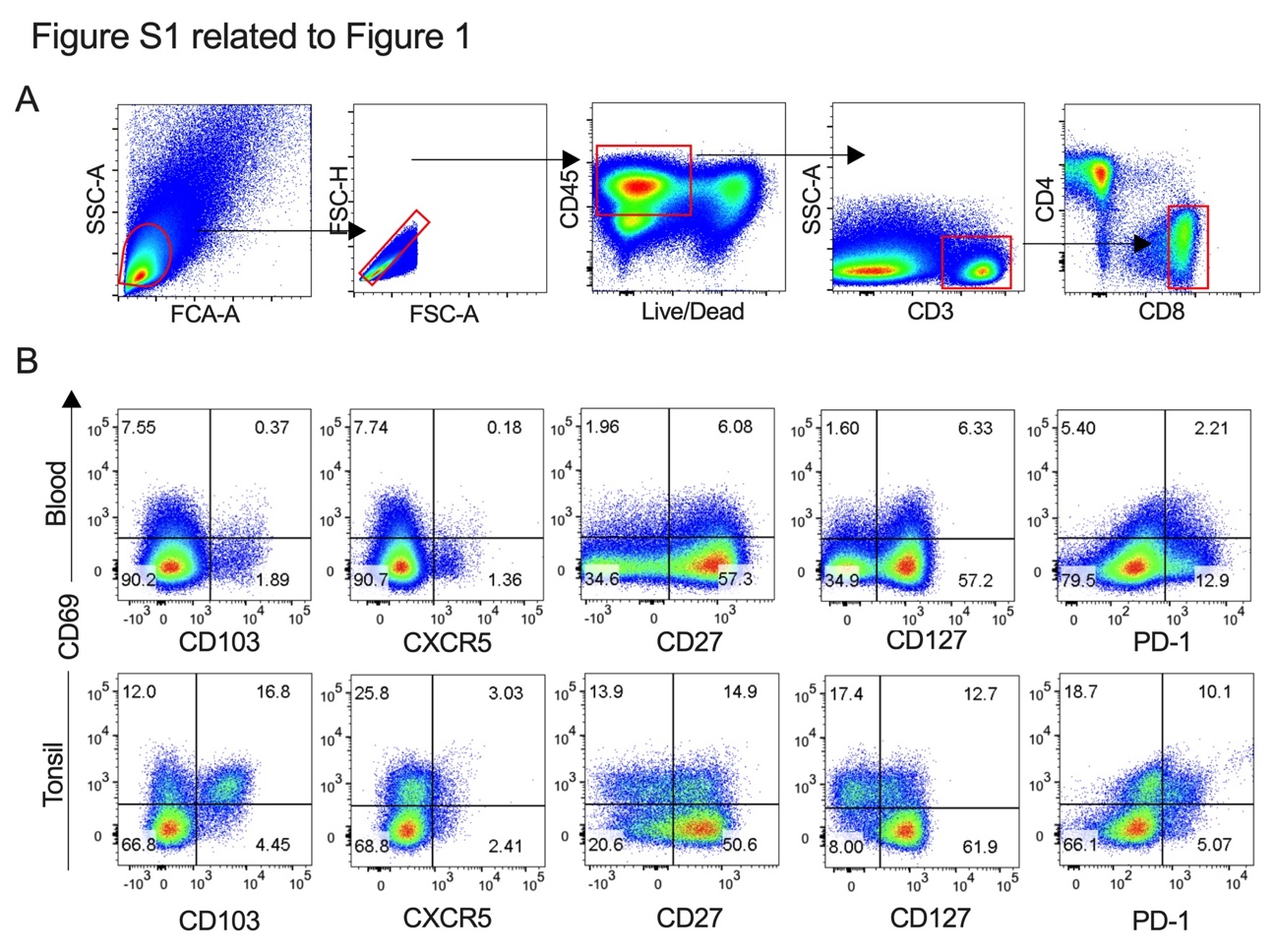
**

**A.** Representative flowcytometry plots (FACS) showing gating of tonsil CD8+ T-cells. **B**. Representative FACS plots of CD8^+^ T cells as CD69^+^ expression versus CD103, CXCR5, CD27, CD127, and PD-1 in blood (top row) and tonsil (bottom row).

**Supplementary figure 2 related to Figure 2.**

**
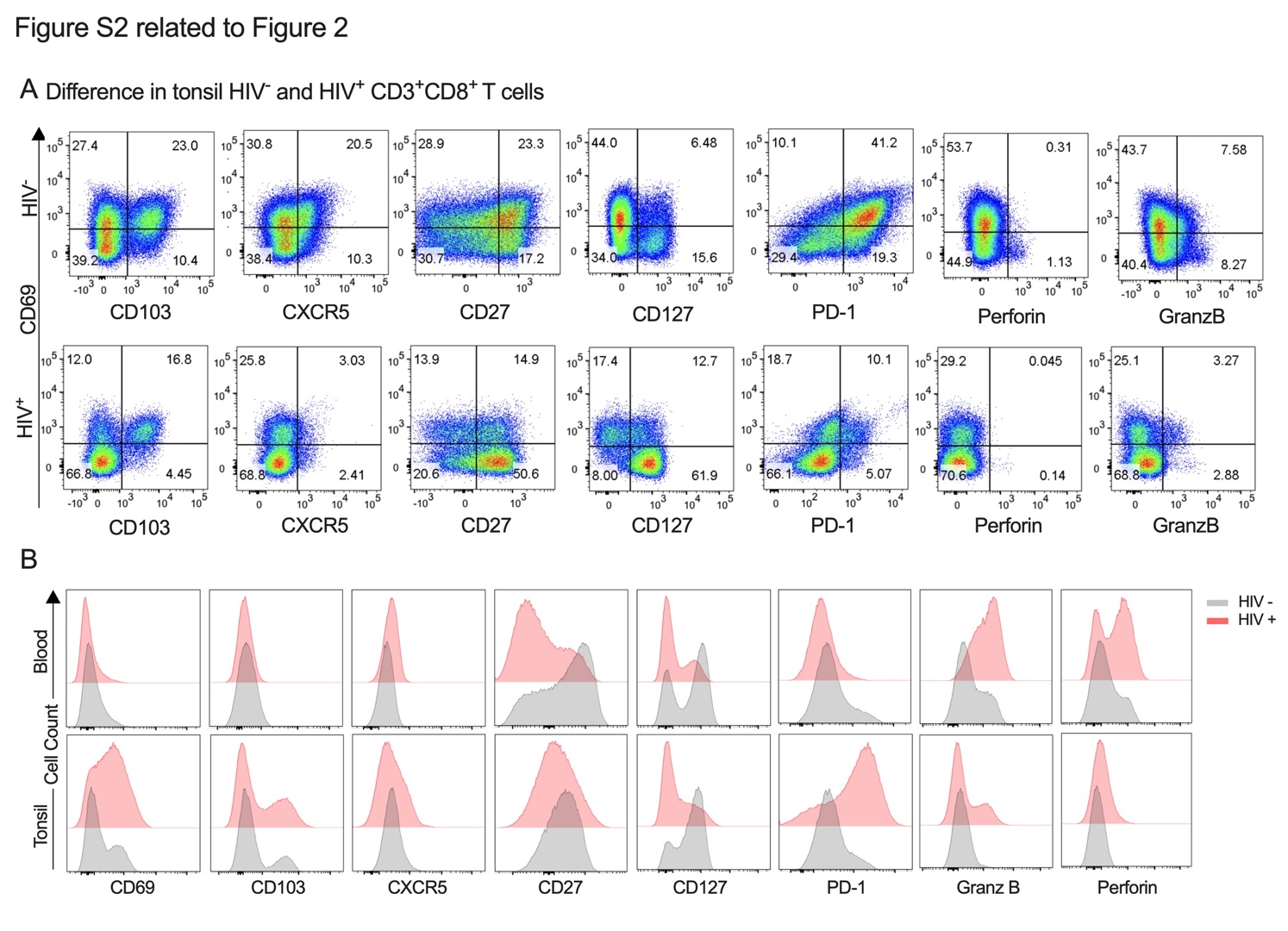
**

**A.** Representative FACS showing gating of tonsil CD8+ T-cells from HIV- tonsil (top) and HIV+ tonsils (bottom) with FACS plots of CD8^+^ T cells as CD69^+^ expression versus CD103, CXCR5, CD27, CD127, PD-1, perforin and granzyme B. **B.** Same as in A but showing histogram overlays for blood (top) and tonsil (bottom) CD8+ T-cells.

**Supplementary Figure 3 related to Figure 3.**

**
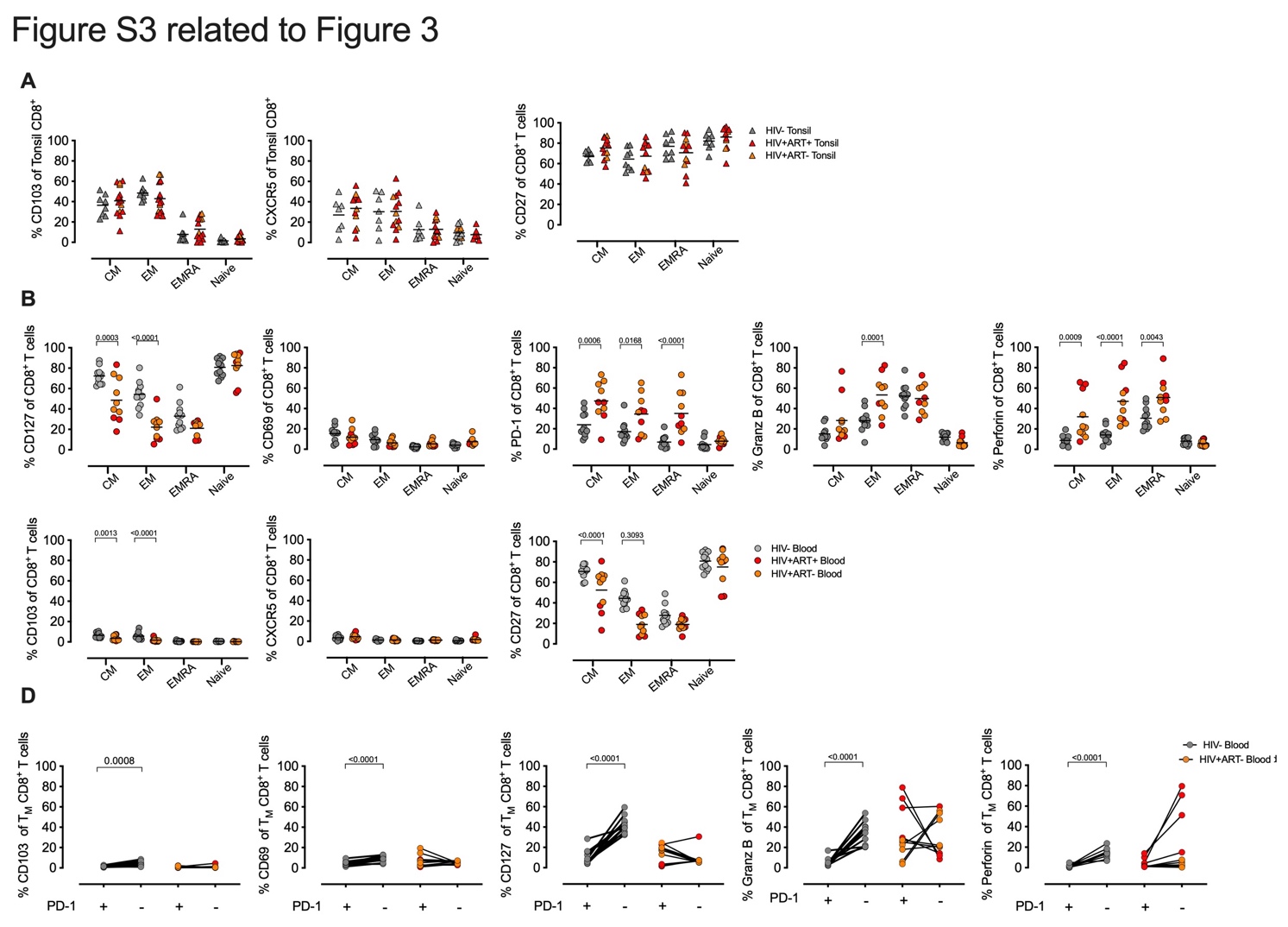
**

**A.** Distribution of blood (*n= 21) central* memory (T_CM_), transitional memory (T_EMRA_), effector memory (T_EM_) and naïve subsets within CD127, CD69, PD-1, granzyme B, CD103, CXCR5 and CD27 and perforin expressing CD8^+^  T-cells cumulative for all study participants in HIV^-^ (grey), HIV^+^ART^+^ (red) and HIV^+^ART^-^ (orange). **B**. Same as in A but showing data from tonsil CD8^+^ T-cells (*n= 22)* expressing CD103, CXCR5 and CD27. P-values were calculated using ordinary one-way ANOVA with horizontal bars representing median values with the level of significance indicated above. **C.** The frequency of CD103, CD69, CD127, perforin, and granzyme B (Granz B) cells was measured on PD-1^+^ (left) and PD-1^-^ (right) CD8^+^ T-cells from blood in HIV^-^ (grey), HIV^+^ART^+^ (red) and HIV^+^ART^-^ (orange) individuals. P-values calculated using Paired Student’s t-test. Horizontal bars represent median values.

**Supplementary figure 4 related to Figure 4.**

**
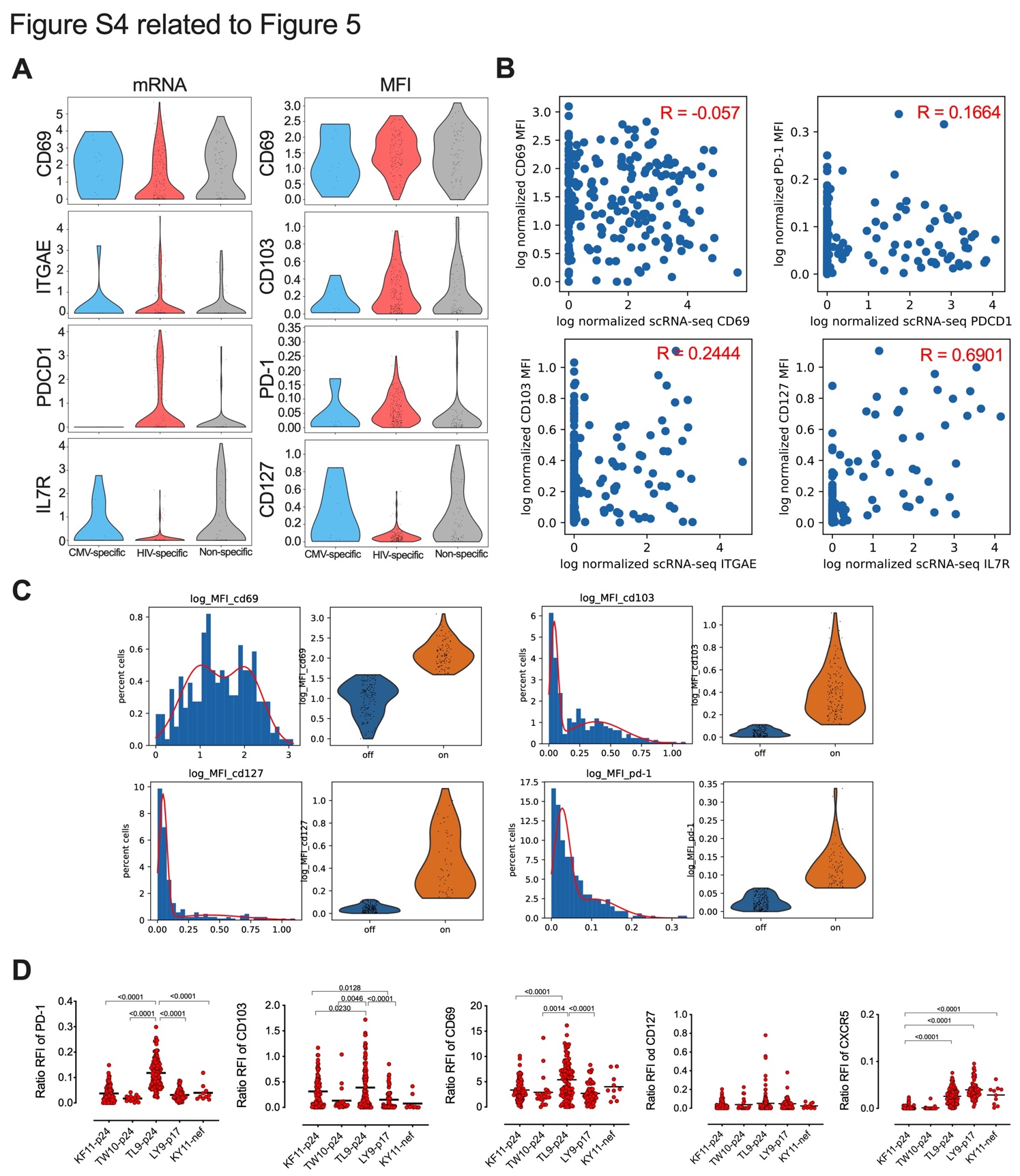
**

**A.** Violin plots displaying mRNA expression from scRNA-Seq (left) and protein matched normalized MFI from flow cytometry (right) for matched single cells for each gene/protein with CMV, HIV and ‘non-specific’ CD8+ T-cells indicated below. **B.** Correlation between mRNA expression from scRNA-Seq and normalized protein MFI from flow cytometry for matched single cells from A. **C.** MFI of histograms and the corresponding violin plots for single cells for CD69, CD103, CD127 and PD-1 expression in HIV-specific CD8^+^  T-cells with on/off indicated and defined as either not expressed (0.0) or expressed (1.0) with cells ordered by high/low normalized MFI Genes ordered by hierarchical clustering. Select genes labelled in plot, full gene lists in Table S4-S7. **D.** Frequency of relative fluorescence intensity (RFI) of PD-1, CD103, CD69, CD127 and CXCR5 in HIV-tetramer specific CD8^+^  T-cells (*n=*3).

**Supplementary figure 5 related to Figure 6.**

**
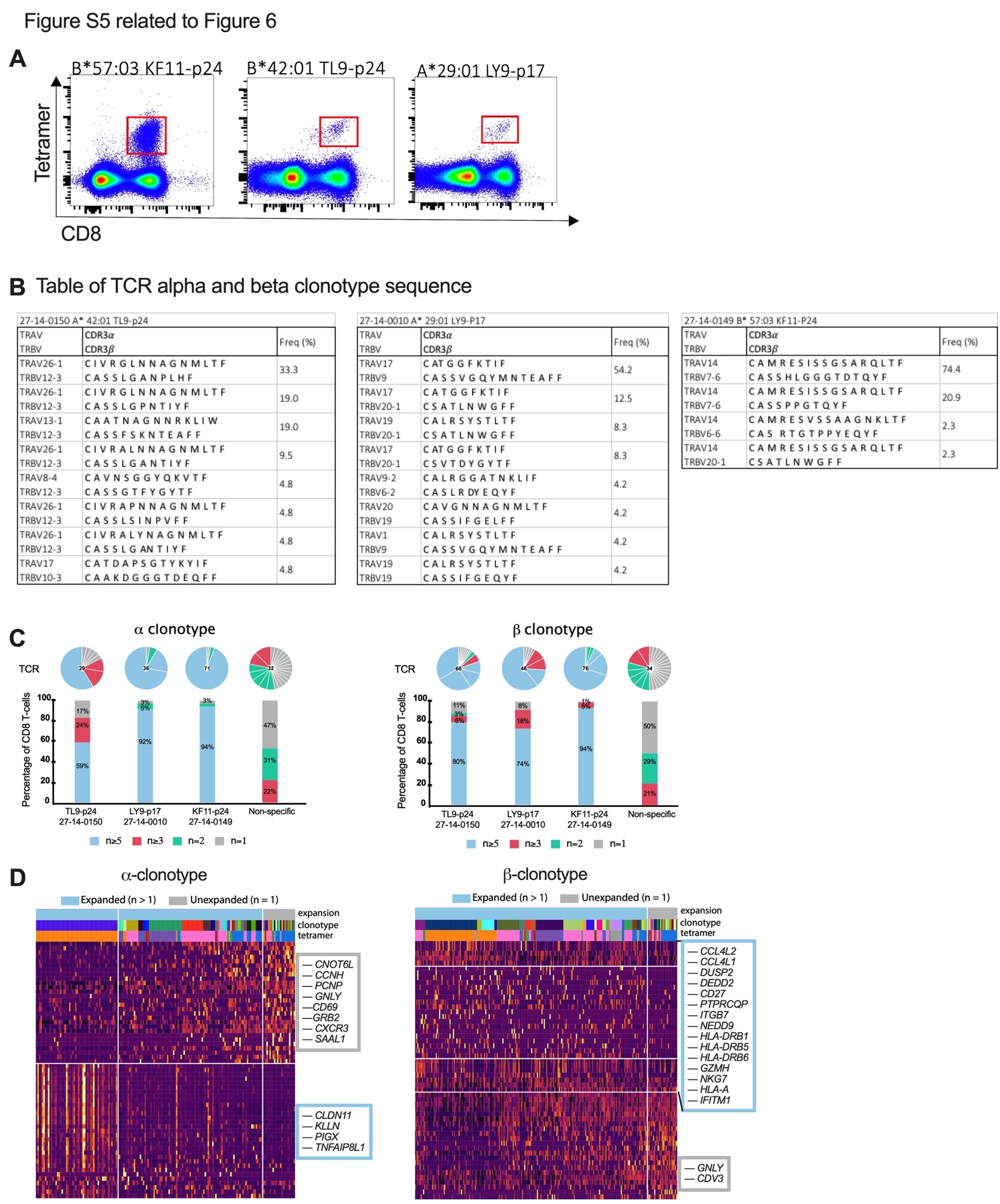
**

**A.** Flow plots showing KF11-p24 (B*57:03), TL9-p24 (B*42:01) and LY9-p17 (A*29:01) tetramer specific CD8+ T-cells in tonsils from 27-14-0149, 27-14-0150 and 27-14-0010, respectively. **B.** TCRα and β clonotype frequency of KF11-p24 (B*57:03), TL9-p24 (B*42:01) and LY9-p17 (A*29:01) tetramer specific CD8+ T-cells. **C.** The TCRα and TCRβ chain distribution of KF11-p24, TL9-p24, LY9-p17 from 27-14-0149, 27-14-0150 and 27-14-0010, respectively or non-specific (Tet^-^) (see Table S2) with unique (n=1), duplicated (n=2), triplet (n ≥ 3) and clonal (n≥5) with bars colored in grey, green, pink and blue representing the fraction of cells belonging to groups of clonotypes with either 1, 2, 3-4 or >5 clonotypes, respectively. Pie charts above each bar illustrate the composition of every individual TCR **D.** Heatmap of z-scored gene expression of top differentially expressed genes (t-test) between Louvain clusters from scRNA-seq data of α clonotype and for β clonotypes**.** Cells grouped by expansion of clonotype, genes grouped by hierarchical clustering. Full gene lists in Table S11 and S12.
